# Nasal Fracture During Maxillary Expansion—A Rare Complication?

**DOI:** 10.3390/reports9020108

**Published:** 2026-04-01

**Authors:** Katharina Obermeier, Wenko Smolka, Philipp Poxleitner, Natasa Puskar, Hisham Sabbagh

**Affiliations:** 1 Department of Oral and Maxillofacial Surgery, LMU University Hospital, Ludwig Maximilian University of Munich, 80337 Munich, Germany; 2 Department of Orthodontics, Clinic of Dental Medicine of Vojvodina, Faculty of Medicine Novi Sad, University of Novi Sad, 21137 Novi Sad, Serbia; natasa.puskar@mf.uns.ac.rs; 3 Department of Orthodontics and Dentofacial Orthopedics, LMU University Hospital, Ludwig Maximilian University of Munich, Goethestrasse 70, 80336 Munich, Germany

**Keywords:** nasal bone fracture, maxillary expansion, orthodontic appliances, removable, cone-beam computed tomography, child

## Abstract

**Background and Clinical Significance:** This case report describes an unusual complication in an 8-year-old female patient undergoing ME (maxillary expansion) with a tooth-supported maxillary expander with the hyrax screw. **Case presentation:** After the 36th screw turn in the 5th week of treatment, the patient reported pressure and pain symptoms and the patient’s parents observed a bone elevation at the bridge of the nose. The patient was referred to for clinical examination which revealed a bilateral infraorbital hematoma and a movable, and highly sensitive, nasal area upon palpation. A cone-beam computed tomography (CBCT) scan confirmed a displaced nasal bone fracture. Conservative treatment was immediately initiated by reversing the hyrax screw four times, followed by ten additional turns over the next 7 days for a total of 14 back-turns. This procedure led to an immediate improvement in symptoms. An 8-week follow-up CBCT confirmed the physiological repositioning of the nasal bones and healing of the fracture. **Conclusions:** Although nasal bone fracture is a rare complication of ME, particularly in children, clinicians should be aware of this potential risk and remain vigilant for symptoms of high pressure and pain in the orbito-nasal area. If a nasal fracture is suspected during orthodontic treatment, the orthodontist should immediately cease screw activation. In selected cases, careful reversal of the screw, as described in this report, may be considered as a conservative treatment.

## 1. Introduction

Transverse maxillary deficiency is a common skeletal anomaly characterized by insufficient maxillary width [1]. Epidemiological data indicate that this condition affects 10% of the worldwide population [1,2]. Clinically, transverse maxillary deficiency can lead to malocclusion, posterior cross bite, maxillary crowding, breathing pattern anomalies and underdevelopment of the midface [3,4,5,6]. Maxillary expansion (ME) is the established orthodontic therapy for correcting transverse discrepancies. In children and preadolescents, orthodontic expansion using tooth-borne appliances, is typically effective and associated with a low risk of complications [7]. In contrast, adolescents and adults often require bone-borne appliances supported by skeletal anchorage or surgically assisted expansion, because of increasing maturation of the palatal and circummaxillary sutures and increased resistance to expansion [7]. Expansion forces affect not only the maxilla but also other biological tissues such as teeth and adjacent cranial structures. Finite element analyses have shown that stress concentrations can be expected in areas such as the pterygoid plates, the foramina ovale and rotundum, and the superior orbital fissure, regions that may be susceptible to subclinical deformation or microfractures under excessive force [8,9,10]. While ME is considered safe in children, complications involving the nasal skeleton, such as nasal bone fracture or disclocation have been described in ME in adolescents and adults [11,12]. This case report describes an unusual complication involving a displaced nasal bone fracture during ME in a pediatric patient and discusses the clinical management and implications for practice.

## 2. Case Presentation

This case reports was prepared in accordance with the CARE guidelines.

An 8-year-old female patient was referred to the Department of Oral and Maxillofacial Surgery (MFS) and subsequently to the Department of Orthodontics and Dentofacial Orthopedics at the Ludwig-Maximilians-University Munich (LMU). She had previously been treated in a private orthodontic clinic for a transverse maxillary discrepancy with a tooth-supported maxillary expander with a hyrax-screw, banded on teeth 16, 26, 55, and 65.

In the fifth week of expansion, after 34 days of one activation per day, the patient reported increasing pain and pressure in the naso-orbital region. Shortly thereafter, her parents noticed a bony elevation at the nasal bridge after the 36th activation of the hyrax-screw (day 36), corresponding to 7.2 mm of transverse expansion and activation was stopped at that time. Accoring to the parents, the originally planned endpoint of the expansion was 8.0 mm, after 40 turns. Two days later, a bilateral infraorbital hematoma was observed. The patient presented to our department on day 42 after the start of treatment, corresponding to 5 days after symptom onset. There was no history of trauma or systemic disease.

**Figure 1:**
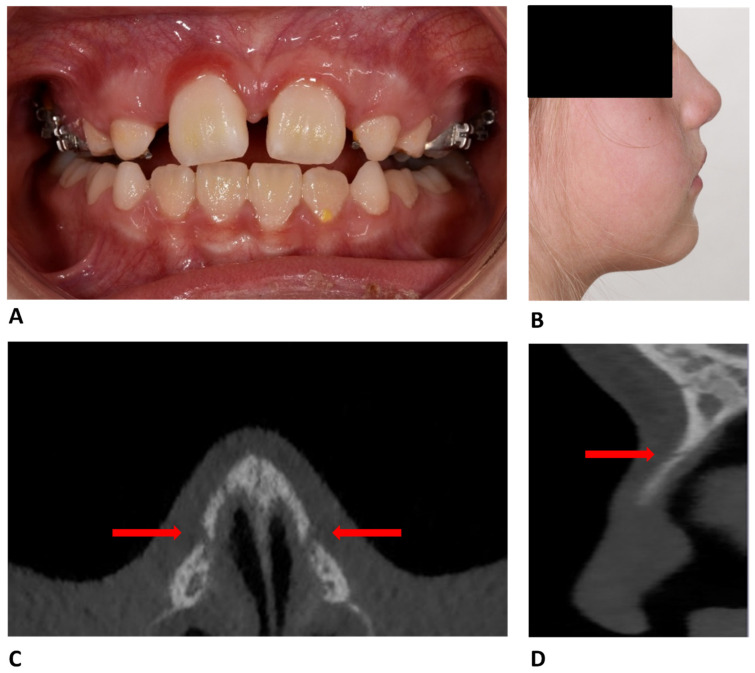
(**A**) Intraoral photograph at the initial visit. (**B**) Extraoral profile photograph. (**C**) CBCT axial slice showing bilateral discontinuity of the nasal bone. (**D**) CBCT sagittal slice showing discontinuity of the nasal bone.

Clinical examination revealed a bilateral infraorbital hematoma, swelling and elevation of the nasal bridge, and an inflamed, slightly mobile, and highly tender nasal area on palpation. Cone-beam computed tomography (CBCT) of the nasal region demonstrated a displaced nasal bone fracture (Figure 1).

Based on the clinical and radiological findings, a diagnosis of nasal bone fracture was made, and conservative management was initiated by reversing the hyrax-screw by four turns at the time of the visit (0.8 mm), followed by an additional ten reverse turns (2.0 mm) at a rate of two back-turns per day. The aim of the partial reverse activation was to relieve symptoms and reduce stress on the fracture while maintaining a sufficient transverse width without lateral crossbite.

**Figure 2:**
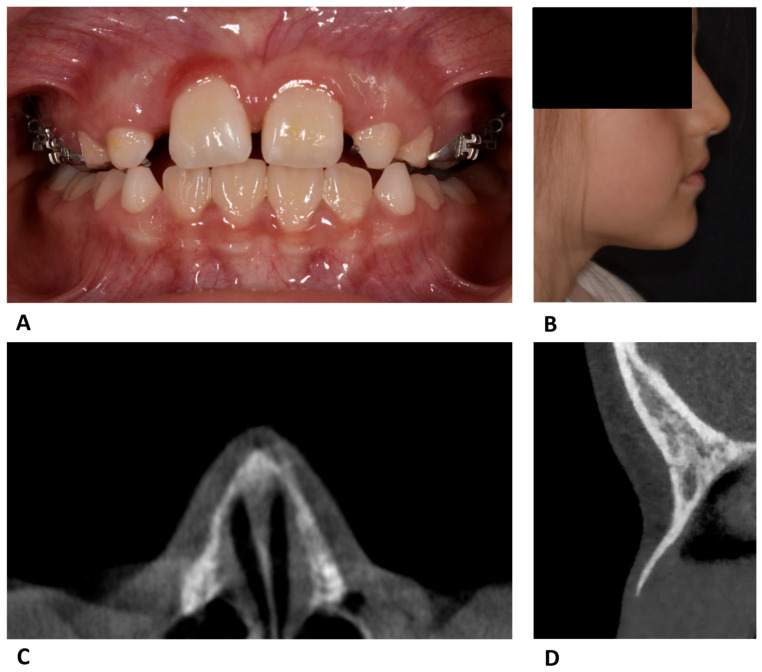
(**A**) Intraoral photograph at the follow-up visit. (**B**) Extraoral profile photograph. (**C**) CBCT axial slice showing bilateral continuity. (**D**) CBCT sagittal slice showing continuity of the nasal bone.

The patient reported immediate relief of pain symptoms after initiating the reverse-activation protocol by four turns. At the follow-up visit after 14 days, reduction in the pressure sensation, infraorbital hematomas, nasal inflammation, and nasal bridge elevation was observed (Figure 2). A CBCT scan obtained after additional eight weeks confirmed a physiological repositioning and healing of the nasal bones (Figure 2).

## 3. Discussion

Maxillary expansion (ME) is a routine treatment modality for maxillary transverse deficiency and can be achieved with different approaches, including conventional tooth-borne appliances, miniscrew-assisted hybrid or bone-borne appliances, and surgically assisted rapid maxillary expansion (SARME) [3,5,8]. In children, tooth-borne ME is generally regarded as safe and effective, with a predominantly orthopedic effect on the midpalatal suture and a low rate of technical and clinical complications. In fact, clinical data indicate that conventional tooth-borne ME produces long-term skeletal effects comparable to those of mini-implant-assisted ME in patients aged 8 to 11 years, with good stability 5 years post-expansion [7]. In this age group, skeletal anchorage is therefore usually not required. Beyond this range, however, it remains unclear up to which age conventional tooth-borne ME is predictably safe and effective, and when other modalities such as miniscrew-assisted or surgically assisted approaches should be preferred. Traditional recommendations based on chronological age and generalized statements about midpalatal suture maturation provide only rough guidance and cannot reliably predict the outcome in an individual patient. More recently, CBCT-based diagnostics of midpalatal suture maturation as proposed by Angelieri et al. have been introduced, but this classification is subject to observer variation and the overall reliability of CBCT-based staging has been questioned [13,14,15,16]. Furthermore, other sutures involved in maxillary expansion, such as the pterygomaxillary, nasomaxillary, and internasal sutures, also show considerable interindividual variation in their ossification and maturation timings and degree of interdigitation. Recently, the risk of unwanted craniofacial fractures associated with advanced suture maturation has been emphasized [12]. As a result, although clinicians can choose on treatment strategies on broad categories such as children, pre- and post-adolescents, and adults, orthodontists should remain aware that complications may still occur, including rare events that are usually not observed in younger patients, as demonstrated by this case report and by similar complications described in two seven-year-old patients after ME [17]. The present case report describes a displaced nasal bone fracture during conventional tooth-borne ME in an eight-year-old child, an age group in which an orthopedic approach would usually be considered appropriate and safe. The observed fracture pattern demonstrated a sagittal separation between the nasal bone and the frontal processes of the maxilla, indicating that forces generated during rapid maxillary expansion are not limited to the transverse dimension but may be distributed throughout the midfacial skeleton and adjacent sutural systems, as demonstrated in previous finite element analysis studies [9,10]. In this context, partial reverse activation likely reduced the transmitted forces to the midfacial skeleton and the mechanical tension within the circummaxillary sutures, thereby allowing repositioning of the displaced nasal bone segment and resolution of the symptoms. In contrast to previous reports, the complication was managed conservatively by partially reversing the expansion while retaining both the appliance and part of the achieved transverse dimension to avoid crossbite and complete retreatment. Orthodontists should therefore not only observe occlusal and skeletal changes during ME but also monitor other symptoms, especially regarding pressure or pain in the nasal and orbital area. This vigilance appears even more important in patients with more advanced skeletal maturation.

## 4. Conclusions

In conclusion, nasal bone fracture is a rare but clinically significant complication of maxillary expansion, even in children. Because sutural maturation and craniofacial response are highly variable and current maturation classifications are not completely reliable, no age can be regarded as entirely free of risk. Orthodontists should therefore remain vigilant for pain and pressure symptoms and morphological changes during ME and be aware of possible complications and their potential handling. Conservative management with controlled screw reversal and close follow-up may offer an effective way to treat cases with complications while preserving part of the achieved maxillary expansion and avoid retreatments.

## Data Availability

The datasets generated and/or analyzed during the current study are available from the corresponding author on reasonable request. The data are not publicity available due to privacy issues.

## Abbreviations

The following abbreviations are used in this manuscript:

ME	Maxillary expansion
CBCT	Cone-beam computed tomography
SARME	Surgically assisted rapid maxillary expansion
MFS	Department of Oral and Maxillofacial Surgery
LMU	Ludwig-Maximilians-University Munich
